# Tell me if you prefer bovine or poultry sectors and I’ll tell you who you are: Characterization of *Salmonella enterica* subsp. *enterica* serovar Mbandaka in France

**DOI:** 10.3389/fmicb.2023.1130891

**Published:** 2023-04-06

**Authors:** Madeleine De Sousa Violante, Valérie Michel, Karol Romero, Laetitia Bonifait, Louise Baugé, Agnès Perrin-Guyomard, Carole Feurer, Nicolas Radomski, Ludovic Mallet, Michel-Yves Mistou, Sabrina Cadel-Six

**Affiliations:** ^1^INRAE, MaIAGE, Université Paris-Saclay, Jouy-en-Josas, France; ^2^ACTALIA, La Roche-sur-Foron, France; ^3^Salmonella and Listeria Unit (SEL), ANSES, Laboratory for Food Safety, Maisons-Alfort, France; ^4^Hygiene and Quality of Poultry and Pork Products Unit, ANSES, Ploufragan-Plouzané-Niort Laboratory, Ploufragan, France; ^5^ANSES, Fougères Laboratory, National Reference Laboratory for Antimicrobial Resistance, Fougères, France; ^6^IFIP–Institut du Porc, Le Rheu, France; ^7^Istituto Zooprofilattico Sperimentale dell’Abruzzo e del Molise “Giuseppe Caporale” (IZSAM), National Reference Centre (NRC) for Whole Genome Sequencing of Microbial Pathogens: Data-Base and Bioinformatics Analysis (GENPAT), Teramo, Italy; ^8^Institut Universitaire du Cancer de Toulouse–Oncopole, Toulouse, France

**Keywords:** *S*. Mbandaka, bovine and poultry sectors, genomic analysis, clone characterization, genomic markers, host adaptation

## Abstract

**Introduction:**

In north-western France, *Salmonella enterica* susp. *enterica* serovar Mbandaka (*S*. Mbandaka) is most frequently isolated from bovine and dairy samples. While this serovar most often results in asymptomatic carriage, for a number of years it has caused episodes of abortions, which have serious economic consequences for the sector. Interestingly, this serovar is also isolated from *Gallus gallus* in the same geographic zone. Despite its prevalence in bovines in north-western France, *S.* Mbandaka has not been broadly studied at the genomic level, and its prevalence and host adaptation are still not fully understood.

**Methods:**

In this study, we analyzed the genomic diversity of 304 strains of *S.* Mbandaka isolated from the bovine and poultry sectors in this area over a period of 5 years. A phylogenetic analysis was carried out and two approaches were followed to identify conserved genes and mutations related to host associations. The first approach targeted the genes compiled in the MEGARESv2, Resfinder, VFDB and SPI databases. Plasmid and phage contents were also investigated. The second approach refers to an in-house algorithm developed for this study that computes sensitivity, specificity, and accuracy of accessory genes and core variants according to predefined genomes groups.

**Results and discussion:**

All the analyzed strains belong to the multi-locus sequence type profile ST413, and the phylogenomic analysis revealed main clustering by host (bovine and poultry), emphasizing the circulation of 12 different major clones, of which seven circulate in poultry and five in the bovine sector in France and a likely food production chain adaptation of these clones. All strains present resistance determinants including heavy metals and biocides that could explain the ability of this serovar to survive and persist in the environment, within herds, and in food processing plants. To explore the wild animal contribution to the spread of this serovar in north-western France, we retrieved *S*. Mbandaka genomes isolated from wild birds from EnteroBase and included them in the phylogenomic analysis together with our collection. Lastly, screening of accessory genes and major variants allowed us to identify conserved specific mutations characteristic of each major cluster. These mutations could be used to design useful probes for food safety surveillance.

## 1. Introduction

Non-typhoidal *Salmonella* spp. is one of the most important foodborne pathogens. The bacterium *Salmonella* is commonly found in the intestines of birds and mammals where it most frequently results in asymptomatic carriage. However, it can cause disease and abortion in bovines, with resulting economic losses (Jawor et al., 2021). In food, the bacterium is mostly found in eggs, dairy products, and raw meat from chickens, turkeys, and pigs. To protect the consumer and to avoid contamination in humans, the European Parliament has implemented regulations that require the control of all foodstuffs and the absence of *Salmonella* in 25 g of test sample. Contaminated animals or food must be destroyed or withdrawn from the market if accidentally already marketed. According to the European Food Safety Authority (EFSA), there were 91,857 confirmed human salmonellosis cases in 2018 in the European Union (EU) (EFSA and ECDC, 2019). The overall economic burden is about 3 billion Euros annually (EFSA, 2014). In France, data collected by the national *Salmonella* Network in 2020 by the French Food Safety Laboratory shows that the sectors most affected by *Salmonella* are the poultry and bovine sectors, and the most frequently isolated serovars are Senftenberg, Montevideo, Livingstone, Dublin, and Mbandaka (*Salmonella* Network Data Report, 2020^1^ and Leclerc et al., 2019). The first four of these serovars have been discussed in the literature (Acosta et al., 2017; Guillen et al., 2020; Sekhon et al., 2020, 2021; Bonifait et al., 2021; Kent et al., 2021), but Mbandaka is a poorly studied serovar even though it is the fifth most frequently isolated serovar in livestock and in the animal production sector in France. *Salmonella* Mbandaka, *S*. Montevideo, and *S*. Dublin account for 66% of the strains isolated in the bovine sector (*n* = 422/639 strains in 2020) (*Salmonella* Network Data Report, 2020)^1^. The *Salmonella* strains collected in the bovine sector originate mainly from samples taken from sick animals and their breeding environment (Peek and Divers, 2016). In adult bovines, clinical signs include fever followed by going off feed, diarrhea with varying amounts of blood, mucus, and shreds of intestinal lining. In milking animals, milk production drops severely (Peek and Divers, 2016). Clinical illness usually lasts for 7–10 days, with recovery in 2 to 3 weeks. Some animals, however, never resume full production. Sick cows that recover may become carriers that shed *Salmonella* for varying periods (e.g., *S*. Typhimurium is shed from 3 to 6 + months, while *S*. Dublin is shed for life) (Kirchner et al., 2012; Holschbach and Peek, 2018). Abortions may occur in infected bovines (Holschbach and Peek, 2018). In north-western France (ISO 3166-2 Metropolitan departments 22 and 35), starting from the spring of 2018, strains of *S*. Mbandaka have also been associated with cases of abortion (personal data). In addition to the economic losses for cow breeders linked to the presence of sick animals on the farm, the presence of *S*. Mbandaka is an additional constraint for producers of milk, dairy products, and cheese in France. According to data from the *Salmonella* Network, 32% (*n* = 480/1,489) of the isolates collected between 2010 and 2020 originated from dairy cows and 14% (*n* = 210/1,489) from milk, dairy products, and cheese (Personal data from the *Salmonella* network, Supplementary Figure 1).

The *S.* Mbandaka serovar is also associated with the poultry sector in France. Since 2010, it has appeared among the top 10 most isolated serovars in poultry from animals and from the production sector. However, it is mainly found in the breeding sector and is rarely isolated from meat and egg products. According to data from the *Salmonella* Network, 98% (*n* = 4,741/4,837) of the isolates collected between 2010 and 2020 comes from animal and poultry breading environment and only 2% (*n* = 96/4,837) from poultry meat and egg products (Personal data from the *Salmonella* network, Supplementary Figure 1). Unlike bovines, poultry are asymptomatic carriers of *Salmonella*. Affected animals may therefore contaminate bovines on mixed farms. Furthermore, recent studies conducted in the United States and Japan on the persistence of *Salmonella* in feces from bovines and various wild animals (crows, deer, feral pigs, raccoons, and waterfowl) have shown that wild animals can also represent a risk of contamination for producers. *Salmonella* can in fact survive in feces for several months and contaminate the farm environment and pastures (Topalcengiz et al., 2020; Yamaguchi et al., 2022).

Certain serovars are able to adapt to several hosts (Stevens and Kingsley, 2021). For example, we have shown that the *S*. Derby serovar isolated in France has adapted to both porcine and *Gallus gallus* hosts, with different genomic clones circulating in the pork and poultry sectors. These clones are characterized by different sequence types (STs): *S.* Derby ST40 is most frequently isolated in the pork sector, and *S.* Derby ST71 in the poultry sector (Sevellec et al., 2018a). The genomic differences between the lineages mainly concern the presence or absence of *Salmonella* pathogenicity islands (SPIs), characteristic substitutions in the *fim*H alleles, and heterogeneity in prophage sequence content (Zheng et al., 2017; Sevellec et al., 2018b).

The aim of this study was to investigate the genomic diversity of the *S*. Mbandaka serovar using a large dataset (*n* = 304) representative of the bovine and poultry production sectors in the north-western production area of France, where this serovar is frequently isolated. The collection was investigated by multi-locus sequence type (MLST) and single nucleotide polymorphism (SNP) analyses. Because resistance of *Salmonella* to antimicrobial agents is a worldwide problem, identification of acquired antimicrobial resistance gene was investigated. We also explored the detection and characterization of SPIs, coding for virulence factors involved in adhesion and invasion of the host, and of the *fim*H gene, known to be a marker of host specificity within *Salmonella*. Genes conferring resistance to heavy metals and biocides, phage sequences, and plasmid content were also evaluated to characterize this poorly studied serovar and to identify potential genome signatures responsible for host specificity of *S*. Mbandaka for bovines and poultry. To increase the chances of finding differences in this serovar’s genome, a host pattern investigation analysis was carried out with an in-house pipeline developed in this study, to identify accessory genes and core variants according to predefined groups of genomes.

## 2. Materials and methods

### 2.1. Strain dataset for France

The panel of 304 *Salmonella enterica* subsp. *enterica* Mbandaka strains analyzed in this study with their metadata (ID, sample name, ID owner, country, collection year, and origin) is presented in Supplementary Table 1 (“Sample DATA” tab). These strains were isolated over a period of 6 years, from 2016 to 2021. Among the panel selected, 140 strains were isolated from the bovine sector and 164 from poultry. All the bovine strains were collected from north-western France, in the *Normandie* region where this serovar is prevalent for bovine sector. The poultry strains, to be compared with bovine strains, were collected mainly (70%) from north-western France to can compare the genomic poultry profiles with the bovine ones but also in the *Pays de la Loire* (*n* = 47), *Bretagne* (*n* = 34), and *Normandie* (*n* = 32) regions. The resting 51 poultry strains were collected elsewhere in France, such as in the *Hauts-de-France*, *Auvergne-Rhône-Alpes*, *Grand Est* or *Occitanie* regions. The number of poultry strains in each region was selected proportionally to its production level compared to the total national production for the poultry sector, with samples isolated both from broilers and layers. One strain of duck and five strains of turkey isolated in north-western France were also included in the panel. The strains from bovine sector come from self-checks carried out at the producers, the ones from poultry sector come from the national surveillance plans carried out for *Salmonella* by the regulation (EC) 2,160/2,003. In both cases, the strains were isolated according to the standard ISO 6579-1 or NF U 47-100 with the scheme of White-Kauffman Le-Minor (Grimont and Weill, 2007).

### 2.2. Sequencing and assembly

All strains were sequenced using Illumina chemistry producing paired end reads as described by Radomski et al. (2019).

Genome assemblies were generated using an in-house workflow called ARTwork (Vila Nova et al., 2019).^2^ The raw reads were normalized to 100× with Bbnorm using the *S.* Mbandaka SA20026234 reference complete genome (NCBI ID CP022489). Then, Trimmomatic (Bolger et al., 2014) was used for the trimming step. The applied quality rules were: (1) length of read higher than or equal to 50 base pairs (bp) otherwise excluded, (2) phred score per base higher than or equal to 30×, and (3) filter away adapters based on an internal database with Illumina adapters. FastQC version 0.11.5 was used to check the read quality and ConFindr to identify intra-and cross-species contamination (Low et al., 2019). SPAdes 3.11.05 was then used to perform *de novo* assembly (Bankevich et al., 2012). Medusa and Gapcloser were used to optimize and finish the assembly (Kremer et al., 2017). Subsequently, QUAST was used to evaluate the quality of *de novo* assemblies by identifying misassemblies and determining error rates (Gurevich et al., 2013). All assemblies were confirmed as belonging to the *S.* Mbandaka serovar with the SeqSero2 tool (Zhang et al., 2019).

Read and assembly quality of the 304 genomes retained in the study is reported in Supplementary Table 1 (“Genome DATA” tab).

### 2.3. Core genome analysis

#### 2.3.1. Multi-locus sequence typing (MLST)

All genomes were characterized by *in silico* MLST using the seven housekeeping gene sequences (*aro*C, *dna*N, *hem*D, *his*D, *pur*E, *suc*A, and *thr*A) described in the PubMLST database (Jolley and Maiden, 2010). The sequence type (ST) of each genome was obtained with the MLST tseeman tool.^3^

#### 2.3.2. Variant calling (SNPs)

The core genome SNPs and small InDels were detected based on the variant caller HaplotypeCaller implemented in the iVARCall2 workflow (Felten et al., 2017). The *S.* Mbandaka strain SA20026234 (accession number CP022489.1) was used as reference complete genome referring to the studies previously published by Cherchame et al. (2022b), and following the best practices proposed by the Genome Analysis ToolKit (GATK) (McKenna et al., 2010). More precisely, secondary alignments around small InDels were performed, and duplications were excluded before variant calling analysis *via* local *de novo* assembly of haplotypes in active regions. The matrices of pairwise SNP differences and pseudogenomes were computed using the scripts called ‘‘VCFtoMATRIX’’ and ‘‘VCFtoPseudoGenome.’’^4^

#### 2.3.3. Phylogenomic inference and calculation of bootstrap support

Phylogenomic inferences were performed by maximum likelihood based on SNP alignment produced by the iVARCall2 workflow and the transversional model TVM + F implemented in the IQ-TREE program (Nguyen et al., 2015). Node support was evaluated with 1,000 rapid bootstrap inferences (Nguyen et al., 2015). Phylogenetic trees were visualized and annotated using interactive Tree Of Life^5^ (Letunic and Bork, 2016).

### 2.4. Accessory genome analysis

#### 2.4.1. Identification of virulence factors and resistance genes

All genomes from France were screened for the presence/absence of genes mediating resistance and virulence using ABRICATE.^6^ ABRICATE was used against the VFDB database (Chen et al., 2016) available at the Institute of Pathogen Biology (Beijing, China), MEGAResV2 (Doster et al., 2020), ResFinder (Zankari et al., 2012), and SPIFinder (Roer et al., 2016), available at the Center for Genomic Epidemiology (CGE) (Denmark). The ABRICATE outputs displayed only the genes detected in at least one genome of the analyzed panel. The threshold was set at 90% identity over at least 3/5 of the length of the gene or genomic region.

Particular attention was paid to the FimH sequence. The *fim*H gene sequence was isolated from the PRJNA297164 project and searched in all 304 *S.* Mbandaka genomes using the BLAST tool (Camacho et al., 2009). All nucleotide sequences found were translated and compared to determine SNP differences between poultry and bovine hosts.

#### 2.4.2. Plasmid identification

All the assemblies were further analyzed to characterize the presence of mobile genetic elements (MGE). MOB-suite tools (Robertson and Nash, 2018) were used to identify plasmid markers. Putative plasmids were blasted against the NCBI nucleotide archive to identify the closest plasmid neighbors.

#### 2.4.3. Phage identification

The presence of phage sequences in the assemblies of French *S*. Mbandaka strains was investigated using the PHASTER online application (Arndt et al., 2016). Only prophages identified as “intact” were considered.

### 2.5. Genome association analysis

Unique markers (genes and variants) and combinations were both investigated to find a genomic pattern of host adaptation (bovine and poultry).

For unique genes, GFF files of all genomes produced by Prokka^7^ were computed by Panaroo (Tonkin-Hill et al., 2020), a graph-based pangenome clustering tool that is able to account for errors introduced during annotation. The clustering identity threshold was increased to 90%. For unique variants, VCF files from iVARCall2 were merged to identify the presence and absence of variants in each genome. The position of variants was identified in *S.* Mbandaka SA20026234. Genes identified by Panaroo or genes containing discriminating variants were annotated using BLAST and UniProt (UniProt, 2021).

To explore possible combinations of markers for host adaptation, we developed a python 3 script called MarkerFinder. MarkerFinder computes the combination of maximum 3 genes or variants with the best discrimination accuracy score according to a phenotypic criterion (in this study, the host origin). MarkerFinder needs two input files: a csv file with the list of genes or variants (produced as described above as outputs by Panaroo and iVarCall2, respectively) and the Excel file compiled with the corresponding phenotype (bovine and poultry in this study). The MarkerFinder output txt file compiles the best combination of maximum three genes or variants found, with accuracy scores, as well as specificity and sensitivity values. The accuracy score was calculated as follow: (TP + TN)/(TP + TN + FP + FN) with TP corresponding to “true-positive result,” TN to “true-negative result,” FP to “false-positive result,” and FN to “false-negative result.” Sensitivity was calculated as TP/(TP + FN) and specificity as TN/(FP + TN). A false-positive result is defined as a positive signal for an identification assay for a gene or variant belonging to a non-targeted phenotype. A false-negative result is defined as a negative result for a gene or variant belonging to the targeted phenotype.

The MarkerFinder tool developed is available at https://github.com/madeleinevlt/MarkerFindr.

### 2.6. Worldwide wild animal genome dataset and phylogenetic analysis

To explore the hypothesis of possible cross-contamination between wild and farm animals, we extracted, from the open access Salmonella EnteroBase database, 2,465 genomes in row reads (on October 2021) whose serovar was confirmed as S. Mbandaka, the MLST profile as ST413, and epidemiologic information-host or country of isolation-available. Among these genomes, 42 were retained because they were isolated from wild animals, with 10 genomes from “Avian,” 2 from “Canine,” 2 from “Marsupial,” 4 from “Reptile,” and 23 “No-determined.” Among these 42 genomes, none were isolated from France or Europe. Finally, considering that only Aves could have been physically responsible for contamination between different continents, we chose nine of the ten genomes from strains isolated from wild birds (“Avian”). One genome was retried due to read errors. Among the final nine genomes selected, six were from coastal regions with five genomes coming from strains isolated along the east coast of North America and one from the west coast. Six genomes were from the United States, two from Canada, and one from Mexico. The accession numbers and epidemiologic information for these genomes are reported in Supplementary Table 1 (“Wild animal DATA” tab).

The variant calling (SNPs) phylogenetic analysis was carried out as described above with the nine genomes from North America and a subset of 214 genomes from France. These 214 genomes (*n* = 100 bovine, *n* = 114 poultry) were selected from the total panel of 304 genomes so that they were representative of the different groups identified by the phylogenetic analysis of bovine and avian strains from France. All singletons were also included (*n* = 41). The S. Mbandaka 100,727 strain (NCBI ID SRR6860551, uploaded at 11/01/2022) belonging to ST506 was also included to root the tree.

## 3. Results

### 3.1. Sequence type genetic diversity and SNP core genome phylogenetic analysis

**FIGURE 1:**
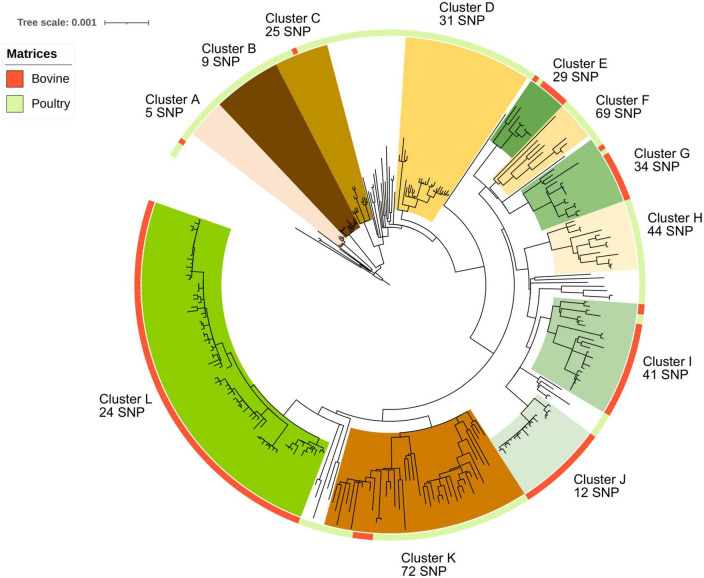
Phylogenetic reconstruction based on core-genome single nucleotide polymorphism substitutions of the 304 *Salmonella* Mbandaka strains isolated in France between 2016 and 2021 from the bovine and poultry sectors. The maximum likelihood criterion and the transversional model TVM + F model were applied. *Salmonella* Mbandaka SA20026234 was used as the reference complete genome. The source of the strains (bovine and poultry) is indicated in the ring around the tree. The 12 genomic groups identified with their intergenomic mean SNP values are indicated by different names and colors for a better visualization.

All 304 *S*. Mbandaka genomes belong to the MLST profile ST413. To investigate the genome diversity of the dataset and identify clustering revealing possible host adaptation, a maximum likelihood phylogenetic tree was built taking into account core genome SNP distances. The tree was rooted using the strain *S*. Mbandaka 100,727 (NCBI ID SRR6860551) belonging to ST506. This ST was identified as genetically closest to ST413 according to hierarchical clustering (HC) cgMLST EnteroBase analysis (Zhou et al., 2020) (data not shown). The phylogenetic analysis revealed 12 major groups supported by 100% bootstrap values and each containing more than five strains. Among these 12 groups, seven groups (A, B, C, D, F, H, and K) contained 118 out of a total of 164 (72%) analyzed genomes sampled from poultry. These seven groups were characterized by genome distances comprised between 5 and 68 SNPs. The other five groups (E, G, I, J, and L) contained 134 bovine genomes over the total 140 bovine genomes analyzed (95%). These five groups were characterized by genome distances comprised between 12 and 34 SNPs (Figure 1). Among poultry samples, 40 did not cluster in any group, while among bovine samples, only 1 genome did not cluster. The maximum SNP distance between all 140 bovine samples was 163 SNPs, whereas all 164 poultry samples had a maximum SNP distance up to 214 SNPs. Interestingly, poultry samples were collected from a wider geographic zone than bovine samples (Supplementary Figure 2). This could explain the higher SNP differences calculated between poultry genomes. Finally, only six poultry samples clustered in bovine groups, while five bovine samples clustered in poultry groups. The six poultry samples clustering within bovine groups were all isolated in the same geographic region (*Normandie*, ISO 3166-2 FR-NOR). Of these six poultry samples, two (S17LNR0975 and S16LNR1497) were genetically close to bovine samples isolated from feces and four (S17LNR0564, S19LNR1916, S18LNR1821, and S17LNR0496) were close to bovine samples isolated from milk. The SNP differences between these samples and the closest bovine samples were comprised between 20 and 38 SNPs. The five bovine samples clustered in poultry groups were all isolated from the same geographic region (*Normandie*). Of these five bovine samples, four (ACT1919926, ACT1919928, ACT1919929, and ACT1919833) were isolated from manure and feces. The last bovine sample (ACT20SMb25) was isolated from bovine feed. The SNP differences between these bovine samples and the closest poultry samples were comprised between 10 and 45 SNPs (Figure 1 and Supplementary Figure 2).

Among the seven poultry groups identified (A, B, C, D, F, H, and K) only group H was characterized by samples isolated from the same region (*Hauts-de-France*). For the bovine groups identified (E, G, I, J, and L), all the samples were isolated in the same region (*Normandie*). Interestingly, in group L, one isolate from cheese (ACT20SMb44) displays only 3 SNP difference with a milk isolate (ACT20SMb64), suggesting likely contamination through the food chain. In the same way, in group J, samples from different matrices (manure, milk, processing plant, and cheese) isolated between 2019 and 2020 clustered together with a mean distance of 12 SNPs (Figure 1 and Supplementary Figure 2).

### 3.2. Accessory genome analyses

The virulome and accessory genome were analyzed to better characterize this serovar and to identify possible genomic differences between strains isolated from the bovine and poultry sectors. Two approaches were followed to identify conserved genes and mutations related to possible bovine and poultry host associations within *S*. Mbandaka strains. The first approach, described in this paragraph, explores the presence/absence of virulence and resistance genes compiled in open access databases targeting known virulence factors, SPI genes, multi-drug resistance genes, and genes conferring heavy metal or biocide resistance. Plasmid and phage sequences were also queried. The second approach, described below, refers to a genomic pattern analysis that computes statistical sensitivity, specificity, and accuracy of accessory genes and core variants according to host associations and specific phylogenetic groups identified.

#### 3.2.1. Virulome analysis

Infection of cells by *Salmonella* requires different steps. After moving to reach epithelial intestine target cells with its flagella, adhesion factors, such as fimbriae and adhesins present on the *S*. Mbandaka surface, enabling strong attachment to the surface of host cells. Gene clusters *csg*, *fim*, *bcf*, and *lpf*, coding for both curli and chaperonne/placier type fimbriae were found in all analyzed strains from France and, although genes coding for SPI-3 were not observed as a whole, *mis*L and *mgt*CB genes coding for adhesins were also observed in the dataset (Supplementary Table 2, “SPI” and “vfdb” tabs). The *fim*H gene was identified in all genomes, and comparison of translated sequences revealed no mutations that could be associated with any bovine or avian host. Only one sequence revealed 1 SNP mutation (i.e., poultry ACT20SMb17 *fim*H nucleotide sequence), but this mutation is synonymous.

Combined analysis of the BLAST results obtained by the SPIFinder and VFDB databases with both thresholds set at 90% identity and 80% identity (data not shown) revealed that SPI-1, SPI-2, SPI-4, and SPI-9, coding for type III (T3SS-1 and T3SS-2) and type I secretory apparatus (T1SS) responsible for survival and proliferation in various intracellular environments, were identified in all the *S*. Mbandaka genomes (Supplementary Table 2, “SPI” and “VFDB” tabs).

SPI-1 genes, encoding T3SS-1 apparatus that is required for invasion of intestinal epithelium cells, such as i*nv, prg*, and *org* gene clusters and *sic*A, *sip*ABCD, *ssp*ABC, *sop*B, *sop*E, and *sop*E2 regulator/effector genes were identified in all genomes. In the same way, SPI-2 genes encoding components of the T3SS-2 apparatus required for bacterial virulence and proliferation in macrophages, such as *ssa*, *ssc*, and *sse* gene clusters and *pip*B, *pip*B2, and *sox*S regulator genes were also identified within all genomes from France (Supplementary Table 2, “SPI” and “VFDB” tabs). Interestingly, the s*ii*ABCDEF SPI-4 gene cluster, encoding a type I secretion apparatus that contributes to the colonization of bovine intestines, was also observed in the dataset, with the exception of five out of the 164 poultry genomes. The few inconsistencies between BLAST results of SPIFinder and VFDB could be explained by the use of different alleles for the same gene in the two databases. It is also important to take into account the identity percentage applied to the analyses. Likewise, only an in-depth analysis of the sequences of these gene clusters within each genome could highlight either SNP or indell type differences between the strains or possible recombination events. Additionally, even though SPI-9 was identified by the SPIFinder database BLAST analysis, operon STY2876, STY2877, STY2878, and STY2875 were not displayed by VFDB BLAST analysis. This inconsistency could be explained by the fact that the ORFs of SPI-9, STY2876, STY2877, and STY2878 present 98% identity with type 1 secretory apparatus (T1SS) genes of SPI-1, inducing likely confusing results. However, further in-depth sequence analyses would be needed to corroborate or rule out this hypothesis (Supplementary Table 2, “SPI” and “VFDB” tabs).

#### 3.2.2. Biocide and heavy metal resistance analysis

At least four of the six genes (*ydd*G, *baeR, bae*S, *sod*A, *rpo*S, and *smv*A) involved in biocide resistance activity were identified within all genomes. Of these six genes, three (*ydd*G, *baeR*, and *bae*S) are described in the literature as involved in methyl viologen dichloride hydrate (paraquat herbicide) resistance (Baranova and Nikaido, 2002; Santiviago et al., 2002). All 140 bovine genomes have these three genes, one poultry genome lacks the *ydd*G gene (S20LNRO591), and one lacks the *bae*R and *bae*S genes (S16LNR1426). The other three genes (*sod*A, *rpo*S, and *smv*A) code for peroxide, hydrogen peroxide, and monochloramine-cation biocide resistances, respectively (Gao et al., 2019; Wand et al., 2019). All genomes have the *sod*A gene coding for peroxide resistance. Only one poultry genome lacks *smv*A (S20LNR0591) coding for monochloramine, and 14 genomes (7 poultry and 7 bovine genomes) have the *rpo*S gene coding for hydrogen peroxide.

The *Salmonella* iron transporter gene cluster *sit*ABCD, coding for a periplasmic ATP-binding- protein, previously identified in *S.* Typhimurium (Kehres et al., 2002) was also identified in all genomes. Interestingly, in line with iron cytoplasm starvation, the *ent*B gene coding for the *Escherichia coli* catecholate siderophore enterobactin (Pakarian and Pawelek, 2016) was identified in all genomes with 80% identity (data not showed), such as the *fep*CG genes that code for ferric enterobactin transport ATP-binding proteins.

Several heavy metal resistance genes were identified. Genes implied in cobalt/magnesium resistance (*cor*ABCD), gold resistance (*ges*ABC and *gol*ST), arsenic (*pst*B), and copper (*cui*D and *cue*P) resistances were found in all genomes. The *mer*CRT genes implied in mercury resistance were also found in only one strain isolated from poultry (S18LNR1211). This last strain also presents the tetracycline, sulphonamide, and aminoglycoside resistance genes *tet*AB, *sul*1, and *ant*3-D,’ respectively (Supplementary Table 2, “MEGAResV2” tab).

#### 3.2.3. Antibiotic resistance gene analysis

All genomes possessed the cryptic aminoglycoside resistance gene aac(6′)-Iaa (Supplementary Table 2, “ResFinder” tab). The ten poultry samples of genomic group D present the plasmid p15ODMR carrying genes conferring resistance to ampicillin (*bla*TEM-1b), sulfonamide (*sul*2), streptomycin (*aph*(6)-Id), tetracycline (*tet*(A)), and trimethoprim (*dfr*A14). The poultry strain S18LNR1211 presents tetracycline, sulfonamide, and aminoglucoside resistance genes *tet*AB, *sul*1, and *ant*3-D’ (Supplementary Table 2, “ResFinder” and “MOB-suite tool–Plasmid” tabs). Mutations on the topoisomerase genes *gyr*A, *gyr*B, and *par*C were identified by MEGAResV2 analysis within all genomes, but they were not associated with fluoroquinolone resistance (Supplementary Table 2, “ResFinder” and “MEGAResV2” tabs). The phenotype of six strains (2021LSAL06158, 2021LSAL06159, 2021LSAL06162, 2019LSAL01500, 2021LSAL06165, and 2021LSAL06166) were analyzed by antimicrobial susceptibility tests confirming these results (data not shown).

#### 3.2.4. Plasmid analysis

The plasmid identification carried out by MOB-suite allows for identification of the plasmid contents in genomes and their mobility. In all, 36 mobilizable, 23 conjugative, and 159 non-mobilizable plasmids were identified. The p15ODMR plasmid present in ten poultry strains of genomic group D carrying multi-drug resistance genes, was characterized as non-mobilizable missing both relaxase and the *oriT* gene. The non-mobilizable plasmid p-F219, previously associated with the epidemic multi-drug-resistance strain of *S.* Infantis isolated from a small farm in the southern region of Peru in 2017 (Vallejos-Sanchez et al., 2019), was identified in 285 genomes in our panel (122 bovines and 163 poultry). The pSLU-1913 conjugative plasmid previously described in *S*. Montevideo strains was identified in 27 strains in our panel, 18 from the bovine sector and 9 from poultry (Bugarel et al., 2019; Supplementary Table 2, “MOB-suite tool–Plasmid” tab).

#### 3.2.5. Phage analysis

**FIGURE 2:**
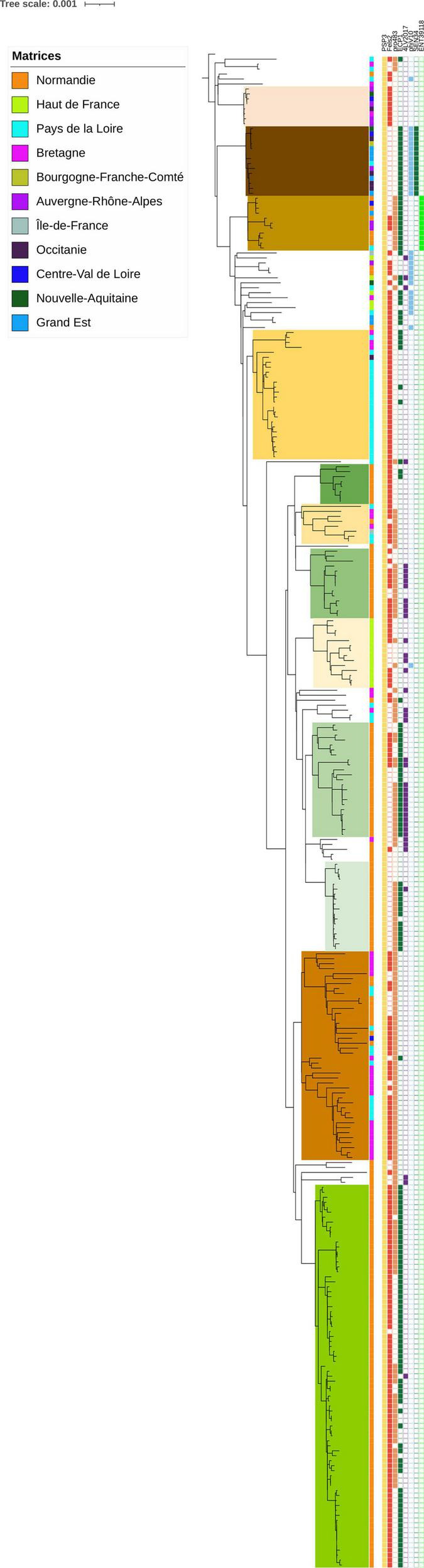
Phylogenetic reconstruction based on core-genome single nucleotide polymorphism substitutions of 304 *Salmonella* Mbandaka strains including the genomic groups identified, geographic origin, and phage profiles. The maximum likelihood criterion and the transversional model TVM + F model were applied. *Salmonella* Mbandaka SA20026234 was used as the reference complete genome.

Eight phages were identified among the *S*. Mbandaka genomes in our panel (Figure 2; Supplementary Table 2, “Phage” tab). One of them, *Salmonella* phage PSP3 was detected in all samples. This phage, belonging to the P2-like phage family, is widely spread within other *Salmonella* serovars (Bullas et al., 1991). The other phages identified were Fels2 (in 207 genomes), pro483 (in 161 genomes), ECP1 (148 genomes), 4LV2017 (41 genomes), phiV10 (29 genomes), SEN34 (14 genomes), and ENT39118 (11 genomes). All these phages are known among *Salmonella* serovars, but some of them such as phiV10 have a high number of genes in common with *E. coli* specific phages (Kim et al., 2017 Phage). Interestingly, the three phages phiV10, SEN34, and ENT39118 were observed only in the genomes of strains isolated from poultry, with the exception of one strain (ACT20SMb25 presenting the phage ENT39118), which was isolated from animal feed on a bovine farm. We did not observe geographic distribution of the phages within the genomes analyzed; however, some of the groups identified were characterized by specific phage profiles such as groups A, D, and E, characterized by phages PSP3 and Fels2, group B characterized by phages PSP3, ECP1, phiV10, and SEN34, or group F characterized by phages PSP3, Fels2, and pro483.

### 3.3. Genes and variants as discrimination markers for hosts

Unique markers and combination of genes or variants were investigated to discriminate host source (bovines and poultry) within our panel of 304 genomes. Any unique marker (gene or variant) was searched among the 5,833 genes and the 3,922 variants identified by the Panaroo analysis. Combinations of genes and variants were identified, allowing us to discriminate the bovine and poultry host source of strains (Table 1). For gene contents, the best combination of genes proposed to discriminate samples from bovines from those originating from poultry hosts was composed of three genes with an accuracy of 0.92, with 91% inclusivity (127/140 bovines identified) and 96% exclusivity (11 false positives) (Table 1).

**TABLE 1:** Duo and trio combinations of genes and variants for host identification (bovine and poultry) within *S*. Mbandaka strains of sequence type ST413 from France.

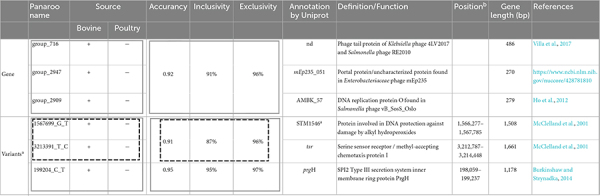		

The duo of genes or variants is framed by a black dotted line as well as the corresponding accuracy, inclusivity, and exclusivity values.

The trio of genes or variants is framed by a gray line as well as the corresponding accuracy, inclusivity, and exclusivity values.

^a^99% identity found.

^b^Reference genome *Salmonella* Mbandaka SA20026234.

nd: Gene name not determined.

The genes identified for the combinations correspond to genes acquired by phages and belong to the accessory genome. For variants content, the best combination identified with two variants has an accuracy of 0.91 and the best combination with three variants displays an accuracy of 0.95, with 95% inclusivity and 97% exclusivity. The three variants identified are in position 199,204, 1,567,699, and 3,213,391 in the genome of *Salmonella* Mbandaka SA20026234. All these variants are located in coding *Salmonella* chromosome sequences. Interestingly, the variant at position 199,204 is located on a gene of SPI2, coding for the type III secretion system protein, which is known to be directly involved in invasion by the bacterium (Jennings et al., 2017). The variant at position 1,567,699 is located on a gene involved in alkyl hydroperoxide resistance, and the variant at position 3,213,391 on a gene coding for a methyl-accepting chemotaxis protein. Further analyses would be needed to determine whether these SNP variations have an impact on the coded proteins.

Finally, with the aim of tracking the *S*. Mbandaka genomic clones belonging to the 12 phylogenomic groups identified in our panel, and without resorting to whole-genome sequencing, we explored the possibility of identifying each group with unique markers of genes and variants (Supplementary Table 3). Using gene content, groups A, B, C, and E can be identified by one unique gene, with 100% inclusivity and 100% exclusivity. Groups F and J can be identified by one unique gene, with 100% inclusivity and 99.7% exclusivity. Group G can be identified with one unique gene with 78% inclusivity and 100% exclusivity. Other groups could not been identified by one unique gene but by unique variants that are able to discriminate these groups and display 100% inclusivity and 100% exclusivity (Supplementary Table 3).

### 3.4. Investigation of wild animal contamination in French herds

**FIGURE 3:**
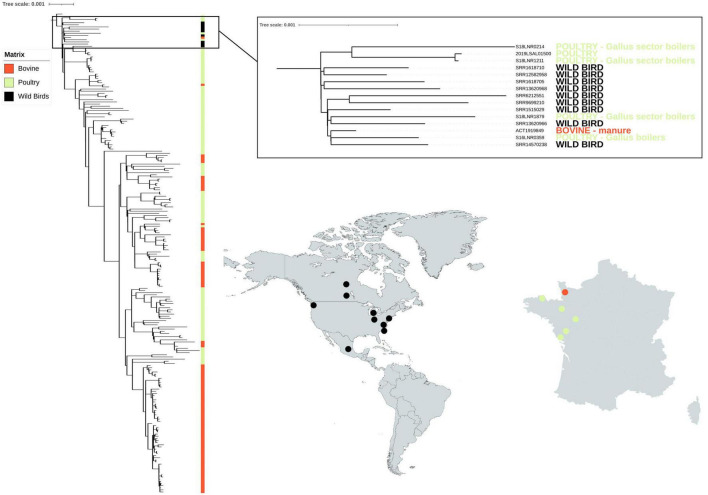
Phylogenetic reconstruction based on core-genome single nucleotide polymorphism substitutions of the 214 *Salmonella* Mbandaka strains from France isolated from the bovine and poultry sectors and nine North American strains isolated from wild birds. The genomes (*n* = 100 bovine, *n* = 114 poultry) were selected so that they were representative of the different groups identified by the phylogenetic analysis of the 304 bovine and avian strains (see Figure 1). All singletons were also included (*n* = 41). The maximum likelihood criterion and the transversional model TVM + F model were applied. *Salmonella* Mbandaka SA20026234 was used as the reference complete genome.

The nine North American genomes from wild birds selected from the *Salmonella* EnteroBase database clustered together with six genomes from France (five poultry samples and one bovine) (Figure 3). Interestingly, the genomes from France were from strains isolated on farms close to the west coast or along a river that flows to this coast. Among the five poultry samples, strains S18LNR1211 and S16LNR3059 were isolated from farms located close to the Atlantic coast in *Bretagne.* Strain 2019LSAL01500 was sampled in the same region and the last two strains S18LNR0214 and S18LNR1879 were isolated further inland, but along a river that flows to the Atlantic coast. Bovine sample ACT1919846 was isolated from manure close to the north-western coast in the *Normandie* region. The mean SNP difference between all these 13 genomes was 57 SNPs (with values ranging from a minimum of 39 to a maximum of 105 SNPs).

## 4. Discussion and conclusion

Phylogenetic analyses carried out on the 304 genomes of *S*. Mbandaka isolated from the bovine and poultry sectors in north-western France revealed strong MLST profile homogeneity. All the bovine isolates belonged to the ST413 profile and, among all poultry strains, isolated in the same period and geographic zone, only five were of different MLST profiles (data not shown). Despite this homogeneity, our panel of strains revealed twelve different clones with a likely degree of adaptation to bovine and avian hosts: 95% of bovine genomes (134/140) clustered into five groups characterized by genome distances of between 12 and 34 SNPs, and 72% of the poultry genomes (118/164) clustered into seven genomic groups characterized by genome distances of between 5 and 68 SNPs. All these twelve groups were supported by 100% bootstrap values. The higher number of genomic groups identified among poultry strains (i.e., 7 poultry groups vs. 5 bovine groups) is likely due to the higher geographic heterogeneity of the samples (i.e., seven different regions versus one for bovine samples) and to the higher number of sample analyzed (i.e., 164 poultry sample versus 140 bovine ones). Given that the samples were isolated over a period of 6 years (between 2016 and 2021) from different matrices, the differences in SNPs observed between the samples within the same group are consistent with those observed for other *Salmonella* serovars such as Typhimurium, Derby or Welikade (Sevellec et al., 2020; Cadel-Six et al., 2021; Cherchame et al., 2022a). The pathways that bacterial pathogens take from farm to fork can be complex, and bacteria can undergo evolutionary changes, especially over long periods. The scientific community agrees that few SNP differences (usually 10 SNPs maximum) closely define related isolates, increasing the likelihood that they arose from the same source (Pightling et al., 2018). The presence of many (hundreds or more) SNPs indicates that isolates are distantly related, implying that they did not originate from the same reservoir population. Interestingly, within bovine strains, in a genomic group identified (i.e., cluster J), only 12 SNPs were recorded between samples from different matrices (manure, milk, processing plant, and cheese) isolated between 2019 and 2020 and only three SNPs were observed between two samples (i.e., cluster L), one from cheese (ACT20SMb44), and one from milk (ACT20SMb64) isolated in 2018 and 2019, respectively. These results suggest an epidemiologic link between these strains and possible contamination/recontamination of the same *Salmonella* clone through this dairy food production chain. This could be due to asymptomatic carriage by animals or use of inappropriate hygiene measures. Within poultry strains, we observed only 5 SNPs between samples belonging to the same genomic group (i.e., cluster A), isolated in 2020 from five different regions (and production). However, it is difficult to interpret these results without having epidemiologic data such as movement of animals between farms, and information on the feed given in breeding or even on the purchase address of primary production chicks and veal.

Nevertheless, what this study shows is that the *S.* Mbandaka clones circulating in France, both in the bovine and poultry sectors, have several multi-resistance genes, likely conferring them the ability to survive several biocides, heavy metals, and drugs. The genomes analyzed carry genes such as *ydd*G, *bae*SR, *omp*D, *smv*A, *par*C, and *mdt*ABCK, conferring resistance to the herbicide paraquat (i.e., methyl viologen dichloride hydrate), peroxide, hydrogen peroxide, and monochloramine (Baranova and Nikaido, 2002; Santiviago et al., 2002). Paraquat, also known under the trade name Gramoxone, was first manufactured and sold by Imperial Chemical Industries in 1962. The European Union authorities banned its use in Court of First Instance of the European Communities (2007) stating that there were indications of neurotoxicity associated with paraquat and a possible link between paraquat and Parkinson’s disease. Peroxide is currently used as a biocide on farms today again. Although there is no standard or protocol imposed by the European Union authorities, peroxide is used in poultry chiller water to reduced aerobic organisms and cross-contamination of poultry carcasses during immersion chilling (Lillard and Thomson, 1983). This compound is also used to clean the claw sleeves between two dairy cows in order to avoid the transmission of mastitis from one cow to another. In general, farm animal drinking water is disinfected with this compound. The advantage of peroxide is that it is used cold and has an immediate effect. Monochloramine is used to disinfect milking machine waters on many farms. Interestingly, it has been demonstrated that the *bae*SR genes are part of a putative operon *mdt*ABCD-*bae*SR that in *Escherichia coli* increases its resistance to novobiocin and deoxycholate (Baranova and Nikaido, 2002). Further experimental tests should be conducted to enable us to assess similar activity in *S*. Mbandaka strains.

This study showed that *S*. Mbandaka can have plasmidic or chromosomic genes conferring resistance to several antibiotics frequently used in the agri-food sector, such as ampicillin (*bla*TEM-1b), sulfonamide (*sul*2), streptomycin (*aph*(6)-Id), tetracycline (*tet*(A and B)), trimethoprim (*dfr*A14), and aminoglycoside (*ant*3-D). Of course, further phenotypical analyses would be needed to confirm the ability of the 304 *S*. Mbandaka strains analyzed to resist paraquat, different biocides, detergents, and antibiotic drugs.

Interestingly, several metal resistance genes were also identified in all genomes conferring to this serovar likely resistance to cobalt/magnesium (*cor*ABCD), gold (*ges*ABC and *gol*ST), arsenic (*pst*B), copper (*cui*D and *cue*P), and iron (*sit*ABCD). Some of these metals, such as copper, are authorized in the European Union as feed additives for breeding and pets animal (Commission Implementing Regulation (EU) 2018/1,039 of 23 July 2018), and are used to treat lameness in dairy bovines (Bolan et al., 2003). Others, such as arsenic, are present in the general environment as a result of human activity such as burning of treated wood (Mandal, 2017) (USGS data 2020). Nonetheless, our results highlight that the evolution of this serovar is driven by anthropogenic selection, as recently observed for other *Salmonella* serovars such as Typhimurium and its monophasic variant *S*. 4,5,12:i:- (Bawn et al., 2020; Cadel-Six et al., 2021).

The *mer*CRT cluster, involved in mercury resistance, was also found in only one strain isolated from poultry (S18LNR1211). This strain also presents the tetracycline, sulphonamide, and aminoglycoside resistance genes *tet*AB, *sul*1, and *ant*3-D,’ respectively. Mercuric resistance was previously described in the epidemic *S*. 4,5,12:i:- clone ST34 associated with resistance to ampicillin, streptomycin/spectinomycin, sulphonamides, and tetracyclines in a composite transposon of the Tn21-family (Petrovska et al., 2016). Similarly, genes conferring resistance to mercury have also been shown in the epidemic *Salmonella* serovar Derby clone ST40 responsible for 70% of human *S*. Derby infections in France (Sevellec et al., 2020). In *S*. Derby, the mercury resistance cluster is located on the transposon of the Tn7-family carried by the *Salmonella* genomic island SGI-1, also encoding the *aadA2* and *sul1* genes mediating aminoglycoside and sulphonamide resistance (Sevellec et al., 2018b). Further tests should be conducted to understand the possible transfer of heavy metal resistance clusters between different *Salmonella* serovars and co-evolution with drug resistance genes.

Gene clusters coding for both curli and chaperon-placier type fimbriae (*csg*, *fim*, *bcf*, and *lpf*) were found in all the *S*. Mbandaka strains analyzed. Interestingly, it was previously shown that *S*. Typhimurium requires Lpf fimbriae for biofilm formation, and other genes such as *mis*L and *mgt*CB involved in biofilm formation (Ledeboer et al., 2006; Wang et al., 2018) that were identified in all *S*. Mbandaka genomes suggesting its likely biofilm formation ability. Finally, despite the analysis carried out on the *FimH* sequence, we were unable to highlight any mutations that could justify speciation to bovine or avian hosts. We were also unable to demonstrate a difference in SPI composition because all genomes analyzed had SPI-1, SPI-2, SPI-4, and SPI-9 coding for type III (T3SS-1 and T3SS-2) and type I secretory apparatus (T1SS), responsible for survival and proliferation in various intracellular environments.

Our genomic analyses revealed that, within their complex, the *S*. Mbandaka ST143 strains analyzed, there was no deep speciation for bovine or avian hosts, but rather different clones circulating within the two sectors. To follow these clones within the different food production chains we identified genomic markers candidate for sub-typing identification analyses. The differences between these clones are manifested by specific genomic variants that rather characterize one clone or the other within one or the other sector. Host pattern analysis carried out allowed us to identify specific sequences for each of the 12 French clusters identified in this study, and a combination of five genes and three variants (i.e., with an accuracy comprised between 0.92 and 0.95) that can identify, within their complex, all the clones belonging to the five bovine phylogenetic clusters circulating in the bovine sector in France. Among the genes, there are some acquired by phages RE2010, mEp235 and vB_SosS_Oslo. Among the three variants, there is a mutation in the sequence of the *prg*H gene that codes for a membrane protein of SPI-2, a mutation in the sequence of the STM1546 gene that codes for a protein involved in protecting DNA from damage caused by alkyl hydroperoxides, and a mutation in the sequence of the *trs* gene cluster that encodes the signaling and chemotaxis complex activated by serine sensing (McClelland et al., 2001; Ho et al., 2012; Burkinshaw and Strynadka, 2014; Villa et al., 2017). Among the genes and variants that are characterized by high performance (i.e., inclusivity 100% and exclusivity comprised between 99.7 and 100%), in each of the twelve phylogenetic clusters identified, six genes and 12 variants were selected. All these sequences constitute ideal sites for the design of genetic markers that could be used for *in silico* or experimental genomic identification analyses such as PCR. There is a clear demand for relevant schemes that are capable of typing and subtyping *Salmonella* and its epidemic clones, ideally with a single technological approach. DNA sequence-based typing methods offer faster, more portable results that allow for a more informative analysis than phenotypic tests or whole-genome sequencing (Wattiau et al., 2011; Bishop et al., 2012). It is clearly useful for producers in the agri-food sector to be able to follow the clones of *S*. Mbandaka throughout the production chain in the event of contamination, but it may also be beneficial to trace these clones in the case of outbreak investigations. Although there have not yet been any associated cases of food poisoning in France, *S*. Mbandaka recently caused human infections in Finland due to contaminated chicken products (FoodSafetyNews, 2022). In 2018, an investigation by the Kalamazoo County Health and Community Services Department and the Michigan Department of Health and Human Services revealed that environmental samples and stool specimens from asymptomatic restaurant employees tested positive for the *S.* Mbandaka outbreak strains (Nettleton et al., 2021). Among the genetic differences that could be used for source tracing, phages such as ΦV10, SEN34, and ENT39118 could also be used. The analysis carried out on the search for phages in the 304 genomes allowed us to highlight that these three phages are present only on clones circulating in the poultry sector. Bacteriophage ΦV10 is a temperate phage, which specifically infects *Escherichia coli* O157:H7 and is closely related to *S.* Anatum (Group E1) phage ∈15 (Perry et al., 2009). Phages SEN34 and ENT39118 were previously described as infecting *Salmonella* strains belonging to *S. enterica* subspecies *enterica*, *salamae*, *arizonae*, *diarizonae*, and *houtenae* (Mikalova et al., 2017; Burnett et al., 2021). It was also previously shown how the presence of phages, such as phage mTmV2, in the *Salmonella* genome constitutes a signature that can contribute to an enhanced ability to track the dissemination of specific clones (Palma et al., 2018).

Geographical segregation and contact with wild animals can also play an important role in the microevolution of emerging clones. In the present study, we tested this hypothesis by focusing on identifying possible wild animal contamination in the geographic and temporal related set of *S*. Mbandaka, all obtained from north-western France between 2016 and 2021. To mine phylogenetic relationships between isolates from different parts of the world, we applied a naïve approach by comparing 214 isolates from France with nine genomes selected from a large set (>2,000) of publicly available genomes of *S.* Mbandaka ST413 isolated from wild animals using the variant calling methodology. The nine North American wild animal genomes selected were all from birds such as gulls, the only wild animals considered able to have entered into contact with livestock animals in France. A recent study by Ahlstrom et al. (2021) provides evidence that gulls can migrate across and between continents and disperse antimicrobial resistant bacteria acquired from anthropogenic sources. In this study, satellite telemetry results of gulls inhabiting Alaska landfills demonstrated autumn migration to Russia, Canada, and California to reach warmer coasts (Ahlstrom et al., 2021). Among the nine genomes of wild birds, six were from the United States, two were from Canada, and one was from Mexico. The isolates were taken over a period of 12 years (between 2007 and 2019) which overlaps with the period of isolation of the French strains. Six French genomes clustered together with these nine North American wild bird genomes, with mean SNP differences of 57 SNPs, a minimum of 39 and a maximum of 105. Interestingly, the mean SNP differences between genomes of the 12 French clusters are comprised between a minimum of 5 and 72 SNPs, with four clusters F, H, I, and K having more than 39 mean SNP differences (i.e., 69, 44, 41, and 72, respectively). The genomes from France that clustered with wild birds were from strains isolated from farms close to the coast or along a river that flows to the coast. The values of SNP differences calculated and the geographic localization of the French samples genomically related to those of wild animals could suggest possible cross-contamination between wild birds and farms. However, further analyses and more genomes such as genomes from wild gulls in France would be needed to confirm this hypothesis. It is known that naturally, the prevalence of *Salmonella* spp. in wild animals is lower than on the farm, despite the fact that wild animals can be asymptomatic carriers of *Salmonella* spp., with the bacterium remaining in equilibrium with the intestinal microbiota (dos Santos et al., 2022). A study carried out on 518 free-living wild animals including mammals, birds, and reptiles in forest fragments in Brazil from 2015 to 2021 showed that only three mammals and one bird were tested positive for *Salmonella* spp (dos Santos et al., 2022). Further investigations would be needed to calculate the frequency of likely contamination of *S*. Mbandaka from wild birds to farm animals in France.

Finally, this is the first genomic study on *S*. Mbandaka. It allowed us to acquire new knowledge about this serovar, both in terms of epidemiology and genomics. The results of the genomic analyses carried out on the 304 *S*. Mbandaka strains underline how the genetic potential of this serovar (i.e., biocide, heavy metal, and drug resistances) could explain why this serovar is present in France in the bovine and poultry sectors. The phylogenetic analyses revealed the presence of 12 major clones, of which seven circulate in poultry and five in the bovine sector in France. The genomic markers study allowed us to determine specific and sensitive sequences that could be used to develop *in silico* or experimental PCRs to trace these clones along the poultry and bovine production chains, or in investigations of foodborne contamination. The MarkerFinder pipeline developed in this study is pathogen-independent and could be used in the future to identify specific and sensitive genes or variants for other foodborne organisms. The 304 genomes that are made available on public databases such as EnteroBase may therefore prove useful for future studies on this poorly referenced serovar. In conclusion, this study has brought to light new aspects of *S*. Mbandaka genomic biodiversity and provides a preliminary overview of the epidemiologic dynamics of this serovar on the farms in north-western France.

## Data availability statement

The datasets presented in this study can be found in online repositories. The names of the repository/repositories and accession number(s) can be found in this article/Supplementary material.

## Author contributions

SC-S, VM, CF, and M-YM: conceptualization. SC-S and MDSV: methodology, formal analysis, data curation, and writing—original draft preparation. SC-S, VM, LBo, and AP-G: strains collection. KR and LBa: experimental analyses. MDSV and NR: bioinformatic development. SC-S, VM, and CF: resources. SC-S, MDSV, VM, AP-G, and LBo: review and editing. SC-S, VM, M-YM, NR, and LM: supervision. All authors contributed to the article and approved the submitted version.

## Footnotes

1
https://www.anses.fr/fr/content/inventaire-des-salmonella-dorigine-non-humaine

2
https://github.com/afelten-Anses/ARtWORK

3
https://github.com/tseemann/mlst

4
https://github.com/afelten-Anses/VARtools/tree/master/iVARCall2

5 iTOL https://itol.embl.de/

6
https://github.com/tseemann/abricate

7
https://github.com/tseemann/prokka

## Supplementary material

Supplementary Figure 1 French *Salmonella* Network data. *Salmonella* Mbandaka data from the French *Salmonella* Network database of the Food Safety Laboratory of the French Agency for Food, Environmental and Occupational Health and Safety (ANSES). **(A)** Distribution of *S*. Mbandaka data from 2010 to 2020. **(B)** Data from *Salmonella* strains isolated from animals and the poultry and bovine production sectors from 2010 to 2020. In the poultry sector, *S*. Mbandaka isolates account for 3,950 entries out of 110,591 (3.5%). In the bovine sector, *S*. Mbandaka isolates account for 1,300 entries out of 17,151 (7.6%).

Supplementary Figure 2 Phylogenetic reconstruction based on core-genome single nucleotide polymorphism substitutions of the 304 *S*. Mbandaka isolated in France from the bovine and poultry sectors, including strain ID, matrix source, and geographic distribution. The maximum likelihood criterion and the transversional model TVM + F model were applied. *Salmonella* Mbandaka SA20026234 was used as the reference complete genome. In all, 12 groups were identified and are highlighted in different colors. Bootstraps comprised between 80 and 100% are shown as triangles at the node’s position.
