# Supplementary figures and images for: Tell me if you prefer bovine or poultry sectors and I’ll tell you who you are: Characterization of Salmonella enterica subsp. enterica serovar Mbandaka in France
Source: Front Microbiol. 2023 Apr 6;14:1130891. doi: 10.3389/fmicb.2023.1130891 (PMC10116068; doi:10.3389/fmicb.2023.1130891)

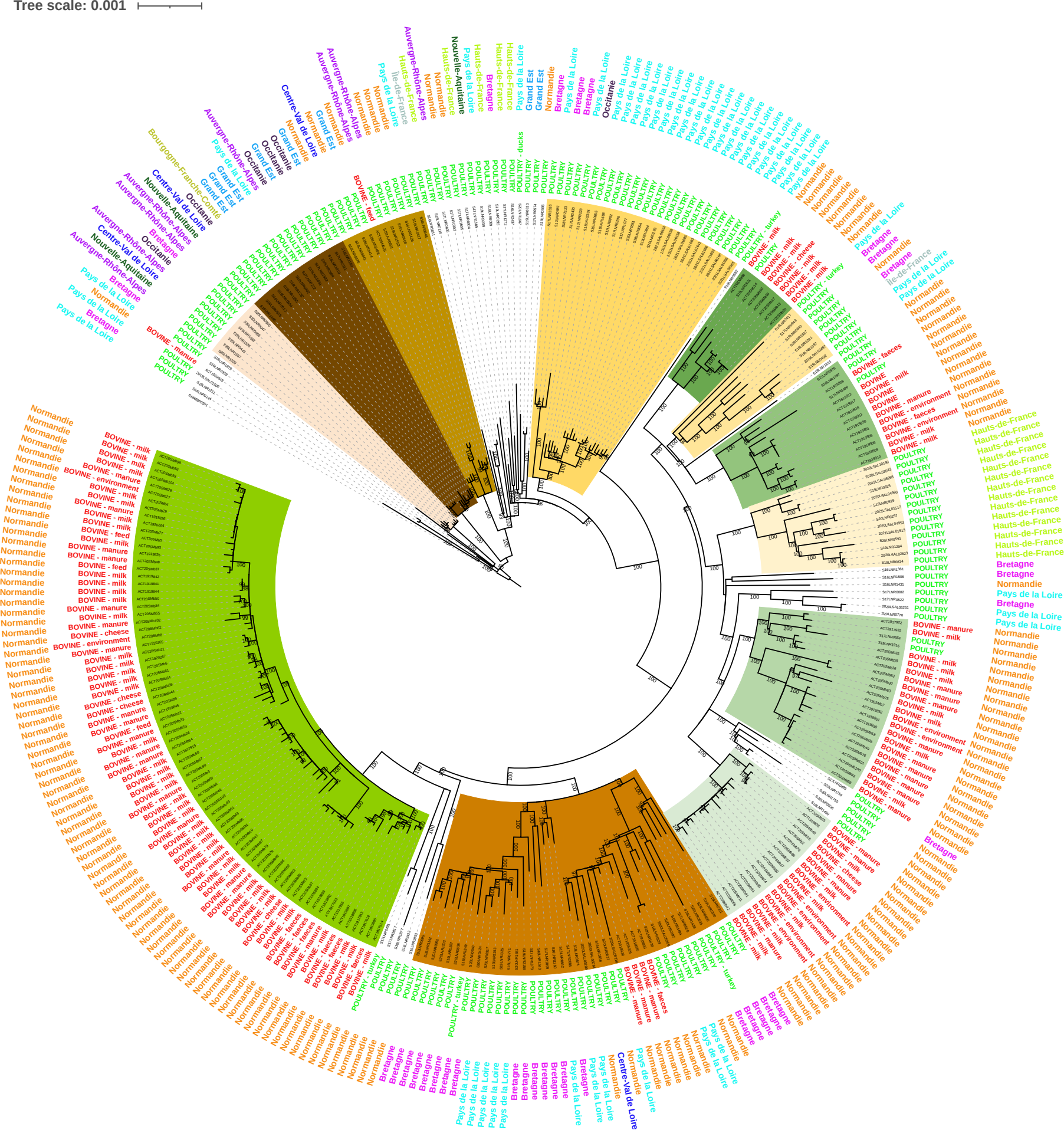

Supplement: Supplementary Figure 1 — French Salmonella Network data. Salmonella Mbandaka data from the French Salmonella Network database of the Food Safety Laboratory of the French Agency for Food, Environmental and Occupational Health and Safety (ANSES). (A) Distribution of S. Mbandaka data from 2010 to 2020. (B) Data from Salmonella strains isolated from animals and the poultry and bovine production sectors from 2010 to 2020. In the poultry sector, S. Mbandaka isolates account for 3,950 entries out of 110,591 (3.5%). In the bovine sector, S. Mbandaka isolates account for 1,300 entries out of 17,151 (7.6%). [file Data_Sheet_1.PDF]

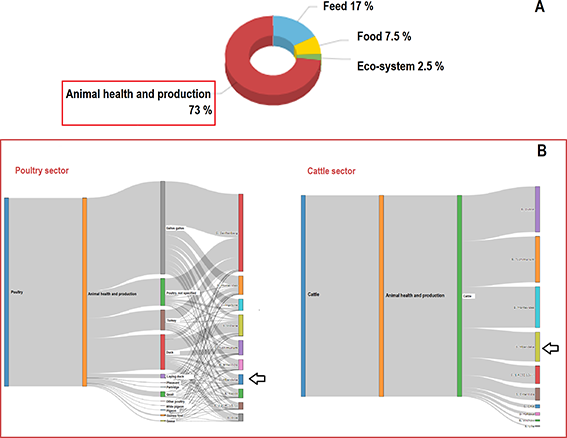

Supplement: Supplementary Figure 2 — Phylogenetic reconstruction based on core-genome single nucleotide polymorphism substitutions of the 304 S. Mbandaka isolated in France from the bovine and poultry sectors, including strain ID, matrix source, and geographic distribution. The maximum likelihood criterion and the transversional model TVM + F model were applied. Salmonella Mbandaka SA20026234 was used as the reference complete genome. In all, 12 groups were identified and are highlighted in different colors. Bootstraps comprised between 80 and 100% are shown as triangles at the node’s position. [file Image_1.TIF]
